# β1,6 GlcNAc Branches-Modified PTPRT Attenuates Its Activity and Promotes Cell Migration by STAT3 Pathway

**DOI:** 10.1371/journal.pone.0098052

**Published:** 2014-05-20

**Authors:** Jingjing Qi, Na Li, Kun Fan, Peng Yin, Chao Zhao, Zengxia Li, Yi Lin, Liying Wang, Xiliang Zha

**Affiliations:** 1 Department of Biochemistry and Molecular Biology, School of Basic Medical Sciences, Fudan University, Shanghai, China; 2 Key Laboratory of Glycoconjugate Research, Ministry of Health, Shanghai, China; 3 Key Laboratory of Molecular Medicine, Ministry of Education, Shanghai, China; 4 Department of Immunology and Microbiology, Shanghai JiaoTong University School of Medicine, Shanghai Institute of Immunology, Shanghai, China; 5 Department of Pediatrics, Affiliated Hospital of Medical College, Qingdao University, Qingdao, Shandong Province, China; University of Liverpool, United Kingdom

## Abstract

Receptor-like protein tyrosine phosphatases (RPTPs) are type I transmembrane glycoproteins with N-glycans whose catalytic activities are regulated by dimerization. However, the intrinsic mechanism involved in dimerizing processes remains obscure. In this study, receptor protein tyrosine phosphatase rho (PTPRT) is identified as a novel substrate of N-Acetylglucosaminyltransferase V (GnT-V). We show that addition of β1,6 GlcNAc branches on PTPRT prolongs PTPRT's cell-surface retention time. GnT-V overexpression enhances galectin-3's cell-surface retention and promotes PTPRT's dimerization mediated by galectin-3. Increased dimerization subsequently reduces PTPRT's catalytic activity on the dephosphorylation of signal transducer and activator of transcription 3 (STAT3) at tyrosine residue 705 (pY705 STAT3), then the accumulated pY705 STAT3 translocates into the nucleus. Collectively, these findings provide an insight into the molecular mechanism by which GnT-V promotes cell migration, suggesting that accumulation of β1,6 GlcNAc branched N-glycans promotes PTPRT's dimerization and decreases its catalytic activity, resulting in enhanced cell migratory capacity.

## Introduction

Glycosylation, known as a common posttranslational modification of glycoproteins,is processed by glycosyltransferases [1],[2]. N-Acetylglucosaminyltransferase V (GnT-V), an enzyme that catalyzes the formation of β1,6 branches of N-acetylglucosamine on N-glycans, plays important roles in carcinogenesis and tumor metastasis [3]. The β1,6 branches of Asn-linked glycans on cell-surface proteins are directly associated with metastasis [4], [5], [6], [7]. Galectin-3 is an endogenous lectin, whose chimera-type domain can bind to polylactosamines [8]. GnT-V-modified β1,6 branches produce numbers of polylactosamines which act as the ligands for galectin-3 with high affinity [9]. By forming molecular lattices with glycoproteins, galectin-3 contributes to cell-membrane retention of vascular endothelial growth factor receptor 2 (VEGFR2), thereby modulating VEGFR2-mediated signals [10].

Protein tyrosine phosphatases (PTPs), as cellular counterpart of protein tyrosine kinases (PTKs), negatively regulate various cellular activities essential for the malignant transformation [11]. There are two kinds of classical PTPs, transmembrane receptor-like PTPs and cytoplasmic PTPs [12]. Receptor protein tyrosine phosphatase rho (PTPRT), a member of type IIB receptor-like PTPs subfamily, normally functions as a tumor suppressor [13]. It has been reported that PTPRT knockout mice are highly susceptible to carcinogen azoxymethane-induced colon tumor [14]. Furthermore, PTPRT dephosphorylates signal transducer and activator of transcription 3 (STAT3) specifically at tyrosine residue 705 (Y705), a phosphorylation site critical for the role of STAT3 [15]. Then, STAT3 phosphorylation at Y705 triggers its dimerization, resulting in the translocation of cytoplasmic STAT3 into the nucleus and transcription of targeting genes associated with tumor metastasis [16], [17], [18].

Although emerging evidences have shown that PTPs play important roles in suppressing tumor progression, the function of aberrant N-glycosylation of PTPs remains to be elucidated. PTPRK (RPTPκ), another member of type IIB receptor-like PTPs subfamily, has been identified as a substrate of GnT-V [19]. Our previous work revealed that overexpression of GnT-V gene in human hepatoma cell line SMMC-7721 induced the addition of β1,6 GlcNAc branches to N-glycans of PTPRK and decreased the phosphatase activity of PTPRK, thus activating EGFR signaling [20]. In the present study, we report that PTPRT can be modified by GnT-V, leading to increased β1,6 GlcNAc branches on PTPRT. Furthermore, overexpression of GnT-V promotes the dimerization of PTPRT in a galectin-3 binding manner and inhibits the phosphatase activity of PTPRT, resulting in heightened phosphorylation level of STAT3 at Y705 which accumulates in nucleus. Activation of STAT3 hence promotes the progression of GnT-V mediated migration. In conclusion, these results reveal a novel role of β1,6 GlcNAc branches on increasing the dimerization of PTPRT and the underlying mechanism of GnT-V mediated cell migration by STAT3 pathway.

## Materials and Methods

### Cell Culture

Cell lines were purchased from Institute of Biochemistry and Cell Biology, Chinese Academy of Sciences. Human GnT-V (NM_002410.3) was cloned into lentiviral vector pCDH-puro. GnT-V overexpressed lentivirus was obtained by collecting supernatant of 293T after cotransfection with envelope, lentiviral construct and packaging vectors (pΔ8.2 and pVSVG) at ratio of 2∶5∶3. HT29 and SMMC-7721 cells were infected with pCDH-puro (Mock) and pCDH-puro-GnT-V (GnT-V) lentivirus and then selected with puromycin at 2 µg/ml. Stable transfectants were maintained in Dulbecco's modified Eagle's medium (DMEM) (Invitrogen, Grand Island, NY, USA) with high glucose or RPMI 1640 medium (Invitrogen, Grand Island, NY, USA) containing 10% fetal bovine serum (FBS) and cultured in a humid incubator at 37°C with 5% CO_2_.

### Nuclear and Cytoplasmic Extract Preparation

Cells were seeded in 10-cm dishes and cultured to confluence. Cells were collected, and washed twice by ice-cold PBS. Cells were suspended in 420 µl of buffer A (10 mM HEPES, pH 7.9, 10 mM KCl, 0.1 mM EDTA, 0.1 mM EGTA) and chilled on ice for 15 minutes. Then, 25 µl of NP-40 (10%) was added, and the suspension was vortexed vigorously for 10 seconds. Cytoplasmic extracts were collected from the supernatants of centrifugation at 15,000 g for 5 minutes. The nuclear pellets were washed with 200 µl of buffer A and suspended in 50∼100 µl of buffer B (20 mM HEPES, pH 7.9, 0.4 M NaCl, 1 mM EDTA, 1 mM EGTA, freshly added protein inhibitor cocktail). The mixture was kept on ice for 15 minutes with frequent agitation. Nuclear extracts were prepared by centrifugation at 15,000 g for 5 minutes. Supernatants were stored at −80°C.

### L-PHA Precipitation

Biotinylated phaseolus vulgaris leucoagglutinin (L-PHA), biotinylated datura stramonium lectin (DSL), agarose bound L-PHA, agarose bound streptavidin and fluorescein anti-avidin D were purchased from Vector Laboratories (Burlingame, CA). Cell lysate was harvested after plating cells in 10-cm dishes and lysing cells with RIPA buffer (1% Triton X-100, 1% deoxycholate, 0.1% SDS) containing protein inhibitor cocktail. Equal amount of cell lysate (800 µg) was precipitated at 4°C overnight with 80 µl of L-PHA agarose. L-PHA precipitates were then centrifuged at 3,000 g and washed three times in ice-cold PBS with protein inhibitors. Precipitates were boiled into sodium dodecyl sulfate (SDS) loading buffer before separated in SDS-PAGE [19].

### Immunoprecipitation

Equal amount of protein samples (1 mg) were incubated with Protein A/G agarose beads (30 µl) (Roche Diagnostics) for 2 hours to clear non-specific binding. After that, lysate was incubated with 2 µg of antibody at 4°C overnight, and then incubated with 30 µl of Protein A/G agarose beads for 4 hours at 4°C. The immunoprecipitates were washed three times with PBS, and separated by boiling in10 µl of SDS sample buffer for 10 minutes. The supernatants were used for followed immunoblot.

### Immunoblot

Anti GnT-V and STAT3 antibodies were from Santa Cruz Biotechnology (Santa Cruz, CA). The PTPRT and galectin-3 antibodies were from Abcam (Cambridge, MA. USA).Cy3-conjugated donkey anti-rabbit antibody and FITC-conjugated goat anti-mouse antibody were bought from Millipore Corporation. Cells were homogenized in SDS lysis buffer on ice, and boiled for 10 minutes. After determining protein concentration with modified Lowry protein assay, equal amount of protein was exposed to SDS-PAGE, followed by electrophoretically transferring to PVDF membrane (Millipore, Saint-Quentin en Yvelines, Belgium) at 300 mA for 2.5 hours in ice. Non-specific binding proteins were blocked by incubating the membrane in 5% fat-free milk or 3% bovine serum albumin (BSA) with TBST. PVDF membrane was then probed with specific primary antibodies overnight at 4°C and the indicated secondary antibodies for 2 hours at room temperature. Proteins were visualized using enhanced chemiluminescence with Image Quant LAS 4000. Protein band intensity was determined by Image J software [19].

### Immunofluorescence and Confocal Laser-scanning Microscopy

Cells were seeded on coverslips in 24-well dishes and cultured overnight. Cells were then washed twice with PBS and fixed with 4% formaldehyde for 20 minutes. After that, cells were blocked with 3% BSA for 2 hours on ice. Cells were stained with anti-PTPRT, anti-galectin-3, anti-pY705 STAT3 or biotinylated L-PHA overnight at 4°C. For secondary staining, Cy3-conjugated anti-rabbit, FITC-conjugated anti-mouse secondary antibody or fluorescein anti-avidin D was used for 6 hours at 4°C. DAPI was used for nucleus staining at room temperature for 30 minutes. Finally, the coverslips were mounted on glass slips with mounting solution. The fluorescence of the cells was visualized by microscopy.

### Cell-surface Protein Cross-linking

Sulfo-NHS-LC-biotin, BS^3^, and protein inhibitor cocktail were obtained from Thermo Scientific. For cell-surface retention assay, cells were washed twice with ice-cold PBS and then incubated with 1 mg/ml sulfo-NHS-LC biotin for 30 minutes on ice. Biotinylation was quenched by addition of 20 mM of Tris-Cl for 20 minutes on ice. Cells were returned to culture for additional 3 or 6 hours [4]. After that, cells were homogenized in lysis buffer and used for immunoblot to detect cell-surface protein level of PTPRT or galectin-3. For BS^3^ cross-linking, cells were exposed to 3 mM BS^3^ for 2 hours on ice and stopped with 20 mM Tris-Cl for 20 minutes on ice. Cells were lysed in SDS lysis buffer for dimerization assay or in RIPA buffer for immunoprecipitaion.

### PTPRT Sample Preparation

Mock, GnT-V cells were lysed in 1×TBS, 1% Triton X-100 supplemented with protease inhibitors. Endogenous free phosphate in these preparations was eliminated before performing the assay through Sephadex G-25 spin columns (Promega). 2 µg of anti-PTPRT antibody was added in each 500 µg pre-cleared lysate for precipitation at 4°C overnight. 50 µl of Protein A/G agarose (Roche) was added to each precipitation at 4°C for 3 hours.

### Tyrosine Phosphatase Activity Assay

The non-radioactive Tyrosine Phosphatase Assay System *in vitro* was performed with the tyrosine phosphatase assay kit (Promega Corporation, Madison, WI, USA) using Tyr phosphopeptide (END(pY)INASL), where pY represents phosphotyrosine as substrate. 96-well dishes containing 0, 100, 200, 500, 1,000 and 2,000 pmol free phosphate and 50 µl of reaction buffer were prepared for a standard curve. Precipitates were washed 3 times in lysis buffer and were subjected to 50 µl of phosphatase buffer (0.9% NaCl, 0.1 mg/ml BSA, 20 mM imidazole, pH 7.2) [21]. Immunoprecipitated PTPRT (10 µl) was then added to reaction buffer as purified enzyme preparations. Phosphatase activity was shown as the level of phosphate released (pmol/min/µl).

### siRNA Transfection

The knockdown of STAT3 and PTPRT was performed using siRNA(synthesized by GenePharma, Shanghai, China).The target sequences were displayed as follows: STAT3 sense, 5′-GGCCAGCAAAGAAUCACAUTT-3′ and antisense, 5′-AUGUGAUUCUUUGCUGGCCTT-3′; PTPRT sense, 5′-GGGCAGCUGACAUUAUUGATT-3′ and antisense, 5′-UCAAUAAUGUCAGCUGCCCTT-3′. Transfection was performed using Lipofectamine 2000 (Life Technologies, Carlsbad, CA, USA) according to manufacturer's instructions.

### Cell Migration Assay

Cell migration was performed using transwell chambers with polycarbonate membrane filters containing 8-µm pore size. 2×10^4^ cells in 200 µl of serum-free medium were placed into each of the upper compartments and medium containing 0.5% FBS was added into the lower chambers as inducer. After incubation for 24 hours, the non-migrated cells were removed with cotton swabs. Cells were fixed with methanol for 20 minutes and stained with crystal violet for 1 hour. Four random fields (100×) were counted per well and the mean was calculated.

## Results

### β1,6 branches of N-glycans on PTPRT are increased in GnT-V overexpressed cell lines

**Figure 1:**
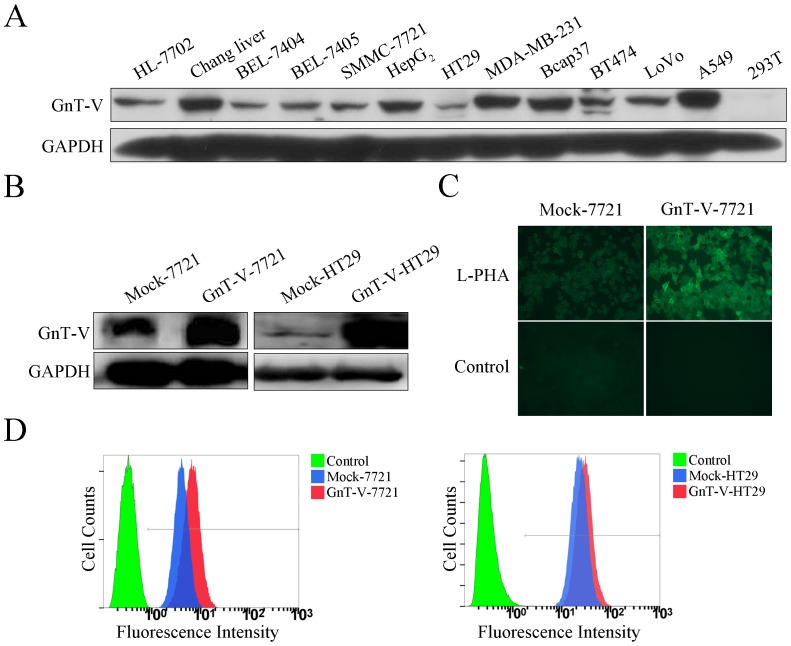
Overexpression of GnT-V causes aberrant N-glycosylation in the transfected cells. (A) The level of GnT-V protein was detected in different cell lines by immunoblot. (B) The GnT-V-7721 or GnT-V-HT29 stable cells were identified by detecting the level of GnT-V protein using immunoblot. (C) GnT-V-7721 and Mock-7721 cells were seeded on coverslips, followed by fixation and staining with biotinylated L-PHA and FITC-conjugated avidin, and then visualized under fluorescence microscopy. (D) Flow cytometry was performed with biotinylated L-PHA in GnT-V-7721 and GnT-V-HT29 stable cells, as well as Mock cells. FITC-conjugated avidin D staining was used as negative control.

In order to establish cell lines with GnT-V overexpression, the GnT-V level was examined in thirteen cell lines. GnT-V protein bands in SMMC-7721, HT29, BEL-7404, HL-7702 cells were slight apart from HepG_2_, MDA-MB-231, A549, Bcap37 (Fig. 1A). Then, we chose cell lines named SMMC-7721 and HT29 for further overexpression of GnT-V gene since their endogenous native GnT-V expression was relatively lower. We infected SMMC-7721 and HT29 cells with GnT-V expression lentivirus or its lentivirus control, designated as GnT-V-7721 and Mock-7721, GnT-V- HT29 and Mock-HT29, respectively. It was identified that the GnT-V was overexpressed in GnT-V-7721 and GnT-V-HT29 cells compared with Mock-7721 and Mock-HT29 (Fig. 1B). Then, a significant increase of L-PHA (specific for GlcNAc β1,6 branches) binding was observed in GnT-V cells compared with that in Mock cells using fluorescent staining and flow cytometry (Fig. 1C and 1D), indicating that β1,6 branches of N-glycans on whole cell-surface proteins were increased as a result of GnT-V overexpression.

**Figure 2:**
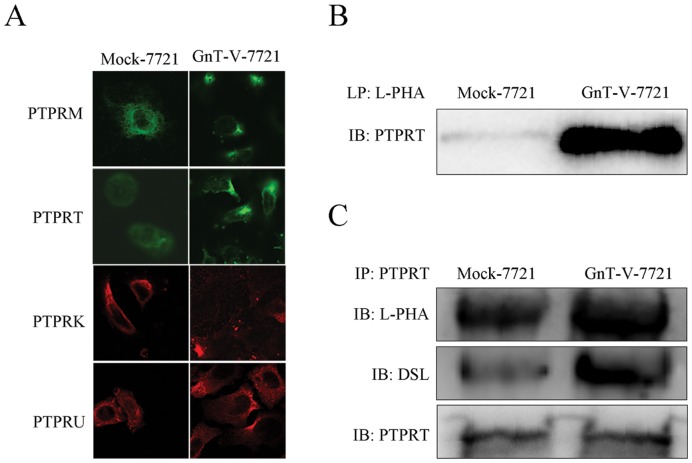
PTPRT is identified as a target of GnT-V. (A) 4 types of RPTPs were evaluated in Mock-7721 and GnT-V-7721 using immunofluorescent staining. Cells were placed on coverslips overnight and stained with anti-PTPRM, anti-PTPRT, anti-PTPRK, or anti-PTPRU antibody, respectively. Then, FITC-labeled anti-mouse IgG or Cy3-labeled anti-rabbit IgG was used based on the specificity of the primary antibody. Intensity of fluorescent was visualized under confocal microscopy. (B) Lectin precipitation was performed with L-PHA bounded agarose, followed by immunoblot with anti-PTPRT antibody in GnT-V-7721 and Mock-7721. (C) PTPRT was immunoprecipitated in GnT-V-7721 and Mock-7721, and then subjected to the immunoblot with biotinylated L-PHA, biotinylated DSL and anti-PTPRT antibody, respectively.

RPTPs are mostly N-linked glycoproteins [13], [19], but the structures and functions of N-glycans in them have not been well understood. To find out a potential target of GnT-V, we investigated the effect of GnT-V overexpression on the cell-surface localization of RPTPs subfamily, PTPRM (μ), PTPRT (ρ), PTPRK (κ) and PTPRU (λ). PTPRT was strikingly increased in GnT-V-7721 cells in contrast with Mock-7721 cells (Fig. 2A). To explore whether PTPRT was a potential substrate of GnT-V, firstly, we predicted its Asn-X-Ser/Thr (NXS/T) sites with NetNglyo 1.0 software and found that PTPRT contained 16 potential N-glycosyslation sites. Then, the lectin L-PHA was employed to precipitate glycoproteins which had β1,6 branches of N-glycans added by GnT-V. The level of precipitated PTPRT exhibited significantly higher in GnT-V -7721 cells than that in Mock-7721 cells (Fig. 2B). Furthermore, by performing immunoprecipitation with PTPRT antibody and then staining with L-PHA or DSL, we confirmed that PTPRT had the structure of β1,6 branches of tri- and tetra-antennary N-glycans (Fig. 2C). Taken together, these results demonstrated that GnT-V overexpression could increase β1,6 branches of N-glycans on PTPRT.

### β1,6 branches of N-glycans on PTPRT are involved in the process of PTPRT dimerization

**Figure 3:**
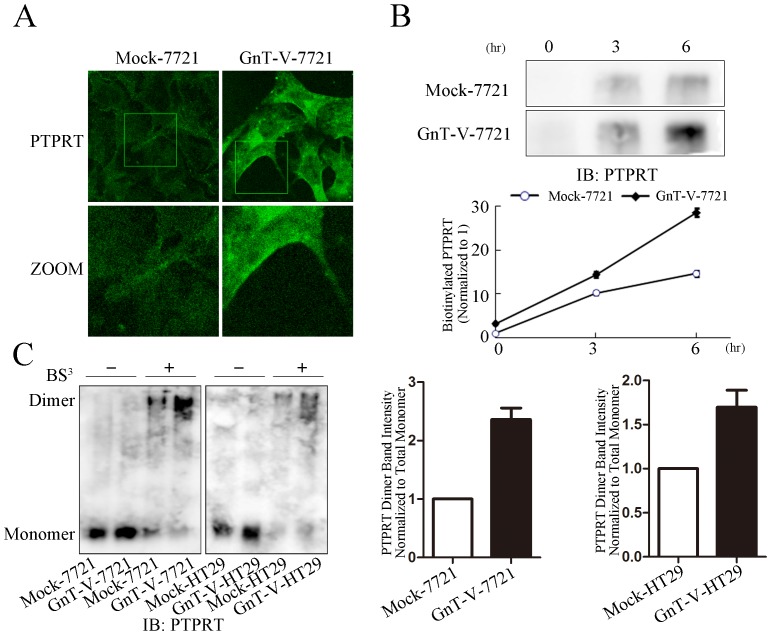
Increased β1,6 GlcNAc branching N-glycans on PTPRT prolongs PTPRT cell-surface retention time and promotes PTPRT's dimerization. (A) Confocal microscopy was performed to observe the distribution of PTPRT in Mock-7721 and GnT-V-7721 cells. The mouse anti-human PTPRT and FITC-conjugated goat anti-mouse IgG were used. The bottom panel represents the magnified images of indicated area in the top panel. (B) Cell-surface retention of PTPRT was examined after cell-surface protein was biotinylated. The stable transfectants were re-cultured for 3 and 6 hours before they were homogenized and pulled down by streptavidin bounded agarose precipitation. After the precipitation, PTPRT was detected by immunoblot using anti-PTPRT antibody. The bottom chart is the quantification of band intensity in the upper panel. The band intensity of PTPRT at 3 or 6 hour time point was normalized to that at 0 hour in both Mock-7721 and GnT-V-7721. The data represent the mean ± SEM of three independent analyses. (C) Mock-7721, GnT-V-7721, Mock-HT29 and GnT-V-HT29 cells were grown to confluence in 6-well dishes and cross-linked with 3 mM BS^3^ at 4°C for 2 hours, followed by immunoblot for PTPRT. The graph (right panel) is the band intensity analysis of dimer band intensity normalized to its total monomer. The data represent the mean ± SEM of three independent analyses.

Alteration of glycosylation on RPTPs could affect their membrane retention level and change their phosphatase activity [20], [21]. We detected the *in situ* PTPRT expression in GnT-V cells and Mock cells. PTPRT obviously accumulated more on cell membrane in the GnT-V cells than that in Mock cells (Fig. 2A and 3A). The cell-surface PTPRT in GnT-V cells and Mock cells was further measured by labeling cells with a cell-impermeatable biotin reagent, followed by re-culturing cells for 3 or 6 hours, precipitating with streptavidin-bound agarose and then blotting with PTPRT antibody. Interestingly, the cell-surface PTPRT was strikingly increased in GnT-V cells in contrast with Mock cells (Fig. 3B). It suggested that β1,6 branches of N-glycans on PTPRT might promote the retention of PTPRT on cell surface. It has been reported that RPTPs clustering at cell surface can mediate their dimerization [4], [21]. To determine whether PTPRT formed dimer on the cell surface, cell-surface protein cross-linking assay was performed in SMMC-7721 and HT29 cells. It was observed that the dimer of PTPRT was more in GnT-V cells than Mock cells, whereas monomer was less in GnT-V cells than Mock cells. The band intensity was analyzed using Image J and the intensity ratio of dimer normalized to momoner was about 2-fold in GnT-V cells of that in Mock cells (Fig. 3C). These results suggested that the increased β1,6 branches of N-glycans promoted PTPRT's dimer formation and increased the amount of PTPRT on cell surface.

### PTPRT's binding with galectin-3 leads to its dimerization

**Figure 4:**
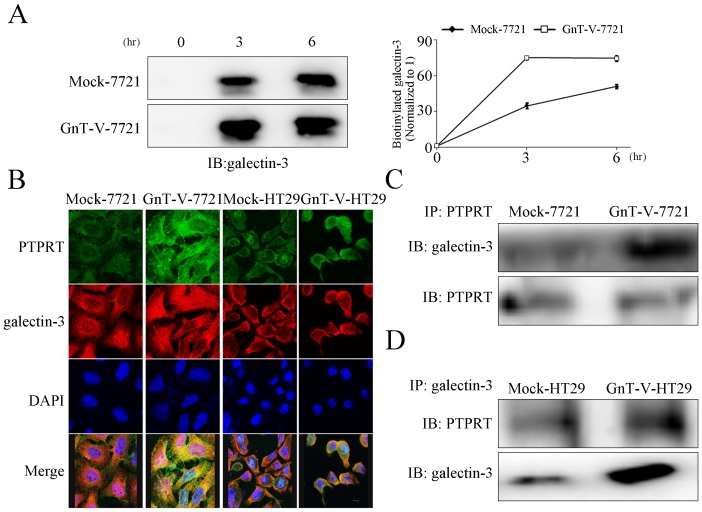
The glycosylation of PTPRT by GnT-V promotes galectin-3 binding, resulting in dimerized PTPRT with increased cell-surface retention. (A) Cell-surface retention of galectin-3 was examined after cell-surface biotinylation. The stable transfectants were re-cultured for 3 and 6 hours before lysis and streptavidin bounded agarose precipitation. After that galectin-3 was detected by immunoblot using anti-galectin-3 antibody. The graph (right panel) is the quantification of the band intensity. The relative amount of galectin-3 at 3 or 6 hour time point was normalized to that at 0 h in both Mock-7721 and GnT-V-7721. The data represent the mean ± SEM of three independent analyses. (B) More colocalization of galectin-3 with PTPRT was visualized at the cell surface in GnT-V overexpressing cells. Confocal microscopy was taken to detect the localization of galectin-3 and PTPRT in Mock-7721, GnT-V-7721, Mock-HT29, GnT-V-HT29. Mouse anti-PTPRT and rabbit anti-galectin-3 were used as primary antibodies. Cy3-conjugated donkey anti-rabbit IgG and FITC-conjugated goat anti-mouse IgG were secondary antibodies. Cell nuclei were visualized by DAPI staining. Merged panels show the overlapped channels. (C) Increased association of galectin-3 with PTPRT is observed in GnT-V overexpressing cells. Mock-7721 and GnT-V-7721 cells were cross-linked with 3 mM BS^3^ at 4°C for 2 hours before immunoprcipitation (IP). IP was performed using anti-PTPRT antibody followed by immunoblot with anti-PTPRT and anti-galectin-3 antibodies. (D) Mock-HT29 and GnT-V-HT29 cells were treated as described in Fig. 4C, except for that anti-galectin-3 was used for IP.

It was found that PTPRT level on cell surface was increased by 2-fold at 6 hour time point after cell-surface biotinylation in the GnT-V cells than that in the Mock cells(Fig. 3B), and the dimerization level was about 2-fold of the Mock cells (Fig. 3C). These data suggested that galectin-3 might exist in the microenvironment which interacted with the β1,6 branches of N-glycans. It has been well known that GnT-V-modified β1,6 branches provide polylactosamines, which are high-affinity ligands of endogenous galectin-3. Therefore, we explored the relationship between PTPRT's binding with galectin-3 and its dimerizaiton. We observed that the cell-surface retention of galectin-3 showed similar tendency to PTPRT in the time course after cell-surface biotinylation (Fig. 4A). The results of confocal laser-scanning microscopy also revealed that GnT-V overexpression led to prominent colocalization of galectin-3 and PTPRT at the cell surface, whereas, in Mock cells, the colocalization was not as much as that in GnT-V cells (Fig. 4B). To identify whether galectin-3 directly interacted with PTPRT, co-immunoprecipitation assay was employed after BS^3^ cross-linking. The results showed an increased binding of galectin-3 to PTPRT precipitated in GnT-V cells (Fig. 4C and 4D). Overall, these results supported the hypothesis that increased N-glycosylation of PTPRT by GnT-V promoted the interaction between PTPRT and galectin-3, which resulted in increased PTPRT cell-surface retention and dimerization.

### Enhanced dimerization of PTPRT in GnT-V cells attenuates its phosphatase activity on STAT3

**Figure 5:**
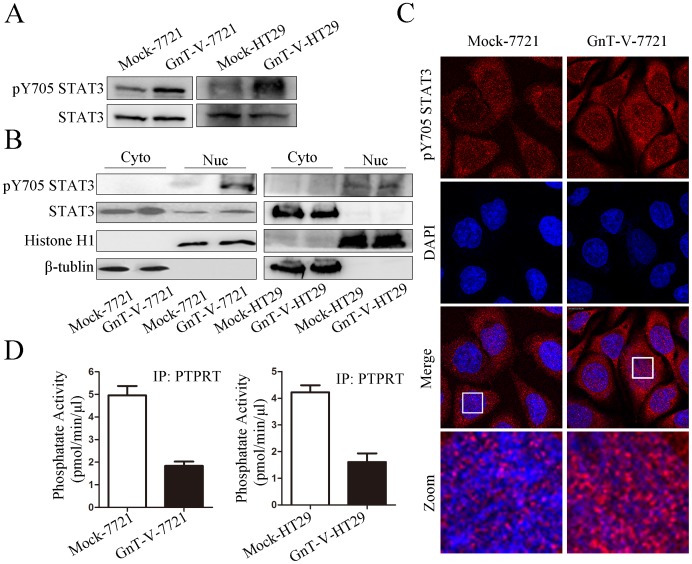
GnT-V overexpression attenuates phosphatase activity of PTPRT, resulting in activation of STAT3. (A) The protein levels of phosphorylated STAT3 at Y705 and total STAT3 were detected using anti-pY705 STAT3 and anti-STAT3 in Mock-7721 and GnT-V-7721, Mock-HT29 and GnT-V-HT29. (B) Cytoplasmic and nuclear fractions were prepared and separated by immunoblot and probed with indicated antibodies. Histone H1 and β-tublin were served as nuclear and cytoplasmic markers, respectively. (C) Subcellular localization of STAT3 in stable transfectants was detected using confocal microscopy. Mock-7721, GnT-V-7721 cells were fixed, permeabilized, and incubated with anti-pY705 STAT3 and Cy3-conjugated secondary antibody. DAPI was used to counter-stain the nuclei. Merged pictures show the overlap of red and blue channels. Zoom, indicated by the white lines, are magnified images of upper panel. (D)Tyrosine phosphatase activity assay was performed in Mock-7721 and GnT-V-7721 (left panel), Mock-HT29 and GnT-V-HT29 (right panel) cells. PTPRT was immunoprecipitated, and phosphatase activity was measured after 15 minutes using tyrosine-phosphorylated peptide as substrate.

As dimerization has been reported to inhibit the activity of RPTPs [22], we hypothesized that the activity of PTPRT might be influenced by its dimerization. To test this, we determined the phosphorylation level of STAT3, a substrate of PTPRT, in Mock and GnT-V cells. Notably, GnT-V cells exhibited higher phosphorylation level of STAT3 at Y705 than Mock cells (Fig. 5A), indicating that PTPRT's dimer form attenuated its activity. It has been reported that phosphorylation at Y705 is critical for STAT3 dimerization, promoting its nucleus distribution and subsequently regulating the expression of its target genes [23]. Accordingly, we observed that the phosphorylated STAT3 at Y705 was mainly accumulated in the nucleus in GnT-V cells compared with Mock cells (Fig. 5B). In addition, compared with Mock cells, the GnT-V cells showed a significant increase of pY705 STAT3 in the nucleus (Fig. 5C). We next examined whether β1,6 GlcNAc branches of N-glycan on PTPRT could influence PTPRT's phosphatase activity *in vitro*. PTPRT sample preparation and phosphatase activity assay were described in Materials and Methods. Results from experiments indicated that phosphatase activity of PTPRT was attenuated more than 50% in GnT-V cells compared with Mock cells (Fig. 5D). The *in vivo* and *in vitro* results indicated that β1,6 GlcNAc branches of N-glycan of PTPRT could inhibit PTPRT catalytic activity.

### Activation of STAT3 is involved in GnT-V promoted cell migration

**Figure 6:**
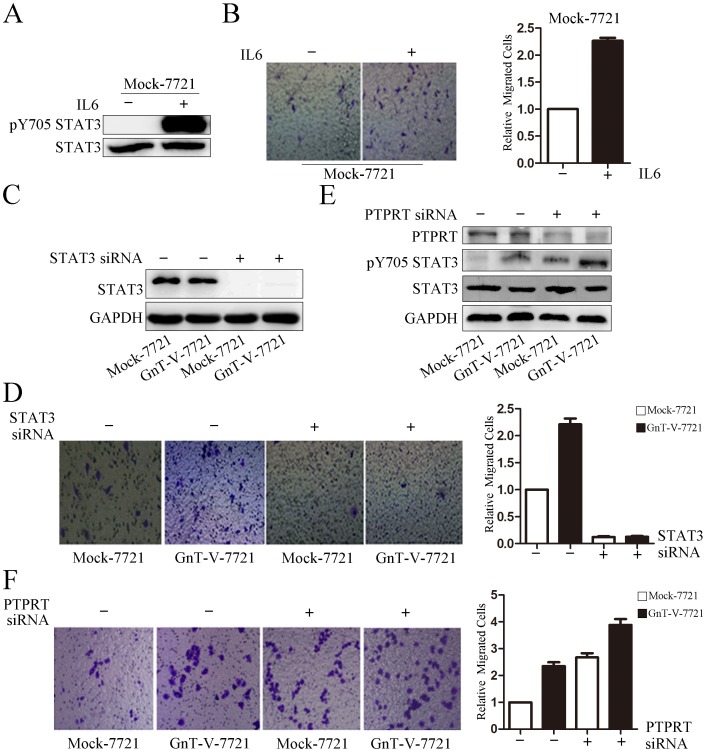
Activation of STAT3 promotes cell migration. (A) Mock-7721cells were treated with IL-6. STAT3 was activated when Mock-7721 cells were treated with 30 µg/ml of IL-6 for 24 hours. The levels of pY705 STAT3 and STAT3 were detected using immunoblot with indicated antibodies.(B) Mock-7721 cells were tested in the transwell assay with or without IL-6 at a concentration of 30 µg/ml for 24 hours. Migrated cells were visualized by staining with crystal violet. The graph (right panel) is the fold changes of migrated cells from the IL-6 treated to the untreated cells. The data represent the mean ± SEM of three independent analyses. (C) Mock-7721 and GnT-V-7721 cells were transfected with synthetic double-stranded control (-) or STAT3 siRNA (+). Two days posttransfection, immunoblot was performed to check the efficiency of knockdown of STAT3. (D) Mock-7721 and GnT-V-7721 cells were transfected with synthetic double-stranded control (−) or STAT3 siRNA (+). Two days posttransfection, transwell assay, described in procedure, was performed to observe cell migration. The graph (right panel) is the fold changes of migrated cells. The data represent the mean ± SEM of three independent analyses. (E) Mock and GnT-V-7721 cells were transfected with synthetic double-stranded control (−) or PTPRT siRNA (+). Two days posttransfection, immunoblot was performed to check the efficiency of knockdown of PTPRT and the level of pY705 STAT3. (F) Mock and GnT-V-7721 cells were transfected with synthetic double-stranded control (−) or PTPRT siRNA (+). Two days posttransfection, transwell assay, described in procedure, was performed to observe cell migration. The graph (right panel) is the fold changes of migrated cells. The data represent the mean ± SEM of three independent analyses.

It has been reported that STAT3 signals could promote cell migration [24], [25], [26]. To explore whether up-regulation of pY705 STAT3 could enhance cell migration, Mock-7721 was treated with IL-6, an activator of pY705 STAT3 [27]. The results showed that pY705 STAT3 level was increased in Mock-7721 cells treated with 30 µg/ml of IL-6 for 24 hours, and cell migration was remarkably promoted in comparison with Mock-7721 cells without IL-6 treatment (Fig. 6A and 6B). We also studied whether GnT-V was involved in cell migration through pY705 STAT3. Cell migration was determined by transwell assay with or without treatment of STAT3 siRNA. Notably, GnT-V cells showed higher mobility compared to Mock cells, and the migrated cells were dramatically decreased in Mock-7 721 and GnT-V-7721 cells when the cells were treated with STAT3 siRNA (Fig. 6C and 6D), implying that STAT3 might contribute to the GnT-V mediated migration. To further evaluate the role of PTPRT in cell migration, immunoblot and transwell assay were determined by knockdown of PTPRT. Immunoblot results showed that the phosphorylation of STAT3 at tyrosine 705 was increased in PTPRT knockdown cells in comparison with negative control cells (Fig. 6E). Then, we found that cell migration was increased significantly when PTPRT gene was knocked down in Mock-7721 and GnT-V-7721 cells (Fig. 6F), probably because of the regulation of PTPRT on the phosphorylation of STAT3. Together, these data indicated that PTPRT's dimer form attenuated its phosphatase activity on STAT3, resulting in pY705 STAT3 accumulation in nucleus, which was responsible for cell migration.

## Discussion

Previous studies have revealed that aberrant N-glycosylation of integrin, EGFR, and N-cadherin modified by GnT-V, resulted in alteration of signal pathways, all contributing to cancer progression [6], [28], [29]. In this study, we find that GnT-V could promote cell migration through PTPRT and STAT3 pathway.

PTPRT belongs to the type IIB receptor-like PTPs and normally functions as a tumor suppresser [14]. The effect of aberrant N-glycosylation in PTPRT molecule on its function has not been well defined. PTPRK, an N-linked glycoprotein, has been reported as a novel substrate of GnT-V [19], [20]. In this study, we are interested in exploring whether GnT-V overexpression could affect PTPRT N-glycosylation and increase the amount of PTPRT at cell surface. PTPRT molecule is predicted to bear 16 potential N-glycosylation sites. Then we use lectin precipitation together with immunoprecipitation experiment to confirm PTPRT as a substrate of GnT-V which provides evidence of the association of GnT-V with PTPRT. We find interestingly that PTPRT accumulates at cell surface in a time-dependent manner, which suggests that there may be a dimerization manner of PTPRT. Moreover, we employ cross-linking assay and immuoblot analysis, which shows a relatively higher dimerization ratio in GnT-V overexpression cells compared with the control cells. Together, these results suggest that GnT-V can add β1,6 branches to PTPRT and promote dimerization of PTPRT.

GnT-V-modified β1,6 branches produce many polylactosamines which are the high-affinity ligands of galectin-3. Evidence shows that galectin-3, with high affinity of GnT-V products, is associated with T-cell receptors (TCR) at cell surface, forming a galectin-glycoprotein lattice that restricts TCR recruitment to the site of antigen presentation [30]. The molecular lattice formed by interaction of galectin-3 and N-glycans modified by GnT-V enhances cell-surface retention levels of epidermal growth factor and transforming growth factor β receptors, which affects their subsequent cell signaling [31]. Based on evidences of interaction of galectin-3 with β1,6 branches, we measure the cell-surface level of galectin-3 and detect the association of galectin-3 and PTPRT. Our data indicate that the galectin-3 molecular lattice is at least part for the increased dimerization of PTPRT.

As phosphorylation of tyrosine residues of proteins plays important roles in regulating diverse biological processes directly relevant to cancer, potential kinase-suppressing activity of PTPs arouses great interest [32]. Dimerization of transmembrane receptor-like PTPs can occlude their catalytic domains and attenuate their phosphatase activities [13]. Furthermore, PTPRT can specifically dephosphorylate STAT3 at Y705 [33]. Accordingly, it would be interesting to evaluate the effect of increased PTPRT dimerization on its substrate. Here, we find that the phosphorylation of STAT3 at Y705 is consistently increased in GnT-V overexpression cells compared with Mock cells, due to the decreased phosphatase activity of PTPRT. Furthermore, the phosphorylated STAT3 at Y705 is mainly located in nucleus using nuclear and cytoplasmic protein extraction experiment or confocal laser-scanning microscopy. It indicates that STAT3 is activated in GnT-V overexpression cells compared with Mock cells. It is well known that activated STAT3 can regulate the transcription of its target genes, which play important roles in the processes of oncogenesis including tumor angiogenesis, tumor cell invasion and metastasis [34]. In addition, STAT3 could bind with other transcription factors such as specificity protein 1 (Sp1), regulating a number of pathways important to tumorgenesis [35]. In the present study, we find that pY705 STAT3 is related to cell migration (Fig. 6A and 6B). The result of migration assay shows that GnT-V cells have higher mobility compared with mock cells. Meanwhile, the migration cells are dramatically decreased in Mock-7721 and GnT-V-7721 cells when the cells are treated with STAT3 siRNA (Fig. 6D). However, the question remains to be explored that whether PTPRT acts as a critical regulator in cell migration through STAT3 phosphorylation. To address this question, we perform the PTPRT knockdown experiment and find that the silence of PTPRT gene increases the level of pY705 STAT3 and promotes cell migration. Therefore, it is reasonable that the β1,6 branches on PTPRT modified by GnT-V play an important role in regulating the STAT3 function. Our findings support that the increased phosphorylation of STAT3 at Y705 might be involved in GnT-V mediated migration.

We conclude that addition of β1,6 branches to N-glycans on PTPRT increases PTPRT's binding with galectin-3, resulting in increased PTPRT dimerization and decreased phosphatase activity, and increased pY705STAT3 promotes the cell migration. Our results provide the possibility that blocking the dimerization process or targeting on STAT3 may have therapeutic implication on GnT-V high-expressing cancer.
